# Facile Preparation of Hydrophobic Aluminum Oxide Film via Sol-Gel Method

**DOI:** 10.3389/fchem.2018.00308

**Published:** 2018-07-30

**Authors:** Changqing Fang, Mengyuan Pu, Xing Zhou, Wanqing Lei, Lu Pei, Chenxi Wang

**Affiliations:** ^1^Faculty of Printing, Packaging Engineering and Digital Media Technology, Xi'an University of Technology, Xi'an, China; ^2^School of Mechanical and Precision Instrument Engineering, Xi'an University of Technology, Xi'an, China

**Keywords:** aluminum oxide film, magnetic stirrer, hydrophobicity, nanowire structures, electroless deposition

## Abstract

Hydrophobic aluminum oxide films (AOFs) are widely used in anti-oxidation and anti-corrosion applications. In preparing AOFs, complex and high temperature conditions are usually necessary. Here, we report aluminum nanowire structures with hydrophobic properties, prepared using a facile sol-gel method by magnetic stirrer and hydrothermal reaction. The electromagnetic force work has great influence on the structure of AOFs. The surface morphology and compositions of the AOFs were analyzed by scanning electron microscope (SEM), energy dispersive X-ray spec-trometers (EDS), X-ray diffraction (XRD), 3M peeling test, and X-ray photoelectron spectroscopy (XPS). With the increase of water content in hydrothermal reaction, the hydrophobicity of AOFs proportional increased. Adding 10 ml deionized water leads to the formation of the upper nanowires and the lower nanohole with 129.3° water contact angle. Meanwhile, the AOF provide a good substrate for electroless deposition (ELD) of copper (Cu) to achieve a simple fabrication of metal conductor.

## Introduction

Hydrophobic aluminum oxide have great influence on many fields due to its prominent capabilities, such as biocompatibility, high mechanical strength, wear resistance, corrosion resistance, hydrophobicity, and high optical transparency (Kim et al., 2012; Chen et al., 2014; Zechner et al., 2014; Xifreperez et al., 2015). Owing to different surface energy, surface wetting properties can be segmented into different kinds. Water contact angle (WCA) in the range of 10–90° and 90–150°, is defined as the hydrophilic and hydrophobic, respectively (Sia and Guo, 2015). Hydrophobicity is a common interface phenomenon, which is closely related to industrial production and life activities, such as fabric printing and dyeing, packaging (Struller et al., 2014), metal coating, paint flow dry performance, and so on. Considering the hydrophobic wide application of alumina, this property has been studied in anti-corrosion metal coating. Preparing hydrophobic Al_2_O_3_ thin films on steel substrates primely improve steel anti-corrosion performances (Chen et al., 2014). Some reported that the preparation of superhydrophobic Al_2_O_3_ nanoparticles via chemical vapor deposition can be applied on the coatings surfaces of different materials and sizes (Bao et al., 2014). In this study, a promising method is proposed for the preparation of anticorrosive superhydrophobic coatings. Chen et al. have prepared Al_2_O_3_-Al coatings with superhydrophobicity and good corrosion resistance and may be applied to a protective layer for marine infrastructure (Chen et al., 2014).

Many publications have proposed methods for preparing alumina films, e.g., chemical vapor deposition, atomic layer deposition, plasma spraying deposition, plasma-assisted chemical vapor deposition, magnetron sputtering, and sol-gel method (Jiang et al., 2011; Sun et al., 2011; Duan et al., 2016; Jeon et al., 2016; Wei et al., 2017; Ding et al., 2018). Most of the methods involve complex, large-scale instrumentation, high temperature processing. To overcome these shortcomings and make a successful growth of hydrophobic AOFs without limitation of substrates, an easy and low temperature sol-gel and hydrothermal treatment method is considered advantageous. The highly homogeneous surface texture Al_2_O_3_ thin films were fabricated by sol-gel at 500°C from aluminum oxide as raw precursor material (Hu et al., 2016). Zhang et al. have proposed that the Al_2_O_3_ film prepared by sol-gel has beneficial effects on the oxidation resistance of the alloy (Zhang et al., 2008).

Here, we propose a new magnetic stirring drive in sol-gel, which is easy to operate and may hold potentially wide applications compared to the stirring paddle (Fang et al., 2018). AOFs are prepared with hydrophobic and hydrophilic properties on the Si substrates via sol-gel method and hydrothermal reaction in polyphenyl (PPL) hydrothermal synthesis reactor. In this research, the water content during the hydrothermal reaction has impacted on the surface wettability properties of AOFs. With the increase of water content, the hydrophobicity of AOFs proportional increased. The strong adhesion of metal coatings on substrates is obviously important for durability of the coating (Lee et al., 2013; Wei et al., 2015). The mechanical adhesion of AOFs was measured by 3M peeling test. In addition, we report electroless deposition of Cu on the prepared AOFs to fabricate metal conductors with low-cost yet highly conductive (Beygi et al., 2012). The morphology of AOFs determines the integrity of the Cu coating. The samples were detected by SEM, XRD, WCA, XPS, 3M peeling test, and I–V curves.

## Experimental

### Materials

Urea (H_2_NCONH_2_, 99 wt% purity, purchased from Tianli Chemical, Tianjing, China), aluminum nitrate nonahydrate (Al (NO_3_)_3_·9H_2_O, 99 wt% purity, purchased from Tianli Chemical, Tianjing, China), single crystal silicon wafer (Si, 99.9 wt% purity, was cleaned with piranha solution before use, purchased from Turbo Technology, Harbin, China), deionized water (purchased from Tianli Chemical Tianjing, China), heating magnetic stirrer (purchased from IKA, Germany), and PPL hydrothermal synthesis reactor (purchased from Huotong Laboratory, Shanghai, China) were used to fabricate AOFs. Concentrated sulphuric acid (H_2_SO_4_, 98 wt% purity) and hydrogen peroxide (H_2_O_2_, 30 wt% purity) were used for preparation of piranha solution. Ammonium tetrachloropalladate(II) [(NH_4_)_2_PdCl_4_, purchased from Meryer Chemical Technology, shanghai, China]. Sodium hydroxide (NaOH, 99 wt% purity), potassium sodium tartrate tetrahydrate (C_4_H_4_O_6_KNa·4H_2_O, 99 wt% purity), formaldehyde solution (HCHO, 37–40 wt% purity) and copper (II) sulfatepentahydrate (CuSO_4_·5H_2_O, 99 wt% purity) were used to electroless Cu deposition (purchased from Tianli Chemical, Tianjing, China).

### Aluminum oxide films fabrication

The 10 × 10 mm Si wafers were cleaned by the piranha solution which was prepared by H_2_SO_4_ and H_2_O_2_ at a volume ratio of 7:3, and were boiled in oil bath pot at 100°C for 12 h, and then wash the Si wafers with ethanol for later use. Urea and Al (NO_3_)_3_ with a molar ratio of 5.2:5.8 mmol were put into 20 ml distilled water by magnetic stirring. The reaction was performed at 80°C, the stirring speed of 250 rpm for 1 h, putting a cleaned Si wafer to the solution of 80°C for another 2 h at 150 rpm. After stirred, the Si wafer was filtered out, heated and dried at 100°C for 1 h in a furnace. Next the Si substrate with a thermally dehydrated Al(OH)_3_ film was transferred into PPL hydrothermal synthesis reactor, to which 500 μl, 3, 5, 10 ml deionized water was also added, respectively. The reactions were run at 200°C for 24 h. Finally the Si wafer with AOFs was dried at 100°C for 1 h. The aluminum oxide films (AOFs), 500 μl-AOF, 3 ml-AOF, 5 ml-AOF, 10 ml-AOF, were obtained on Si substrates.

### Electroless Cu deposition

The AOFs were put in the (NH_4_)_2_PdCl_4_ aqueous solution for 15 min in the dark to load ${\text{PdCl}}_{4}^{2-}$ by ion exchange, rinsing with deionized water. The electroless Cu deposition happened in a plating bath being composed of a volume ratio of 1:1 mixture of freshly prepared solution A and B for 10 min. Solution A contains NaOH (12 g dm^−3^), CuSO_4_·5H_2_O (13 g dm^−3^) and C_4_H_4_O_6_KNa·4H_2_O (29 g dm^−3^) in deionized water. Solution B is a HCHO (9.5 cm^3^ dm^−3^) aqueous solution (Wang et al., 2016). Then, rinsing samples with deionized water and drying by air.

### Characterization methods

XRD patterns were recorded on a Shimadzu Limited diffractometer operating at 40 mA and 40 kV using monochromatic Cu Kα radiation (the scan speed of 8.0 deg min^−1^, from 10 to 70°), and it was used to analyze the crystallinity of the AOFs. The morphologies of the AOFs were analyzed by Field Emission Scanning Electron Microscope (FE-SEM, SU8000) at 1 KV with EDS at 20 KV. The WCA was measured on an OCA 20 instrument (Dataphysics, Germany) dropping a volume of 2 μl water on sample surface. XPS studies were performed on an AXISULTRA with a monochromatic Al Kα source to analyze the film composition on Si surface. XPS spectra were emitted photon energy of 1486.71 eV at a power of 100 W (10 kV, 10 mA) with the vacuum about 10^−8^ Torr. The charge neutralizer was used to compensate for surface charge effects, and binding energies were referenced to the C1s hydrocarbon peaks at 284.8 eV. The mechanical adhesion of AOFs was researched by 3 M peeling test. A 12.7 mm-wide piece of 3 M scotch transparent tape (3 M Company, America) was attached on the sample and kept 90° angle peeling off the sample after 2–3 min. The process was repeated 10 times using a new piece of tape for each test and the mechanical adhesiveness was evaluated by measuring the weight loss of AOFs after each tape test. The I–V curves of Cu coatings on AOFs were evaluated with a CHI660E electrochemical workstation.

## Results and discussions

**Figure 1:**
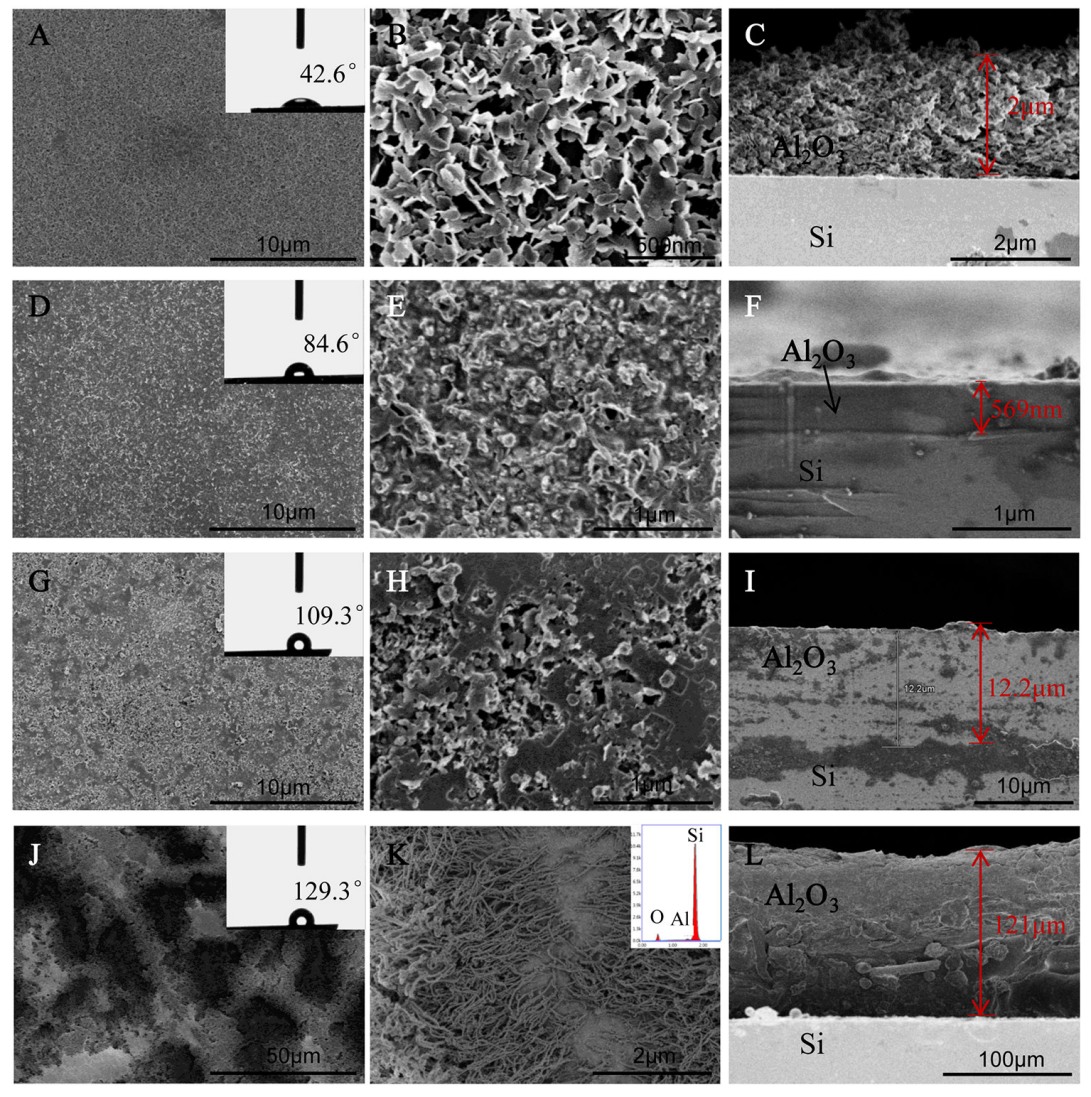
SEM sans of the surface and cross-section of AOFs on Si substrates: **(A–C)** 500 μl-AOF; **(D–F)** 3 ml-AOF; **(G–I)** 5 ml-AOF; **(J–L)** 10 ml-AOF, the inset of K is energy spectrum image. The insets of **(A,D,G,J)** are water contact angles (are the maximum or minimum angle in the all angles to show the significance of hydrophobic or hydrophilic).

The SEM images showed surfaces morphologies of AOFs in Figure 1. The 500 μl-AOF consisted of loose irregular sheet structure and large gaps. The surface roughness increases significantly owing to high surface energy, resulting in small WCA. From the cross-section image, the 500 μl-AOF layer was dense on Si substrate with the thickness of about 2 μm (Figures 1A–C). With the water content increasing in hydrothermal reaction, the SEM images of samples including 3 and 5 ml-AOF films showed the particles were packed tightly and glued together (Figures 1D,E,G,H). In Figure 1F, it was seen that the 3 ml-AOF layer gradually became flat and dense on Si substrate with the thickness of about 1 μm. The thickness of the film was increasing, and 5 ml-AOF layer was 12.2 μm. The water contact angle of films was significantly increased, and it showed an increasing hydrophobicity. When the water content was 10 ml, the film was formed by both the upper nanowires and the lower nanohole, with water contact angle of 129.3°, and was also the topography of hierarchical micro/nanoroughness (Figures 1J,K). In Figure 1L, the film thickness reached 121 μm from cross-section image. The structures, formed by special nanowire clusters, made a signification contribution to reduce the surface energy for achieving the hydrophobic features of the AOFs. It was confirmed that the water content of 10 ml in hydrothermal reaction was sufficient to create a hydrophobic surface with hierarchical micro/nano roughness. The energy spectrum demonstrated that the sample composition was O, Al, and Si in the inset of Figure 1K.

**Figure 2:**
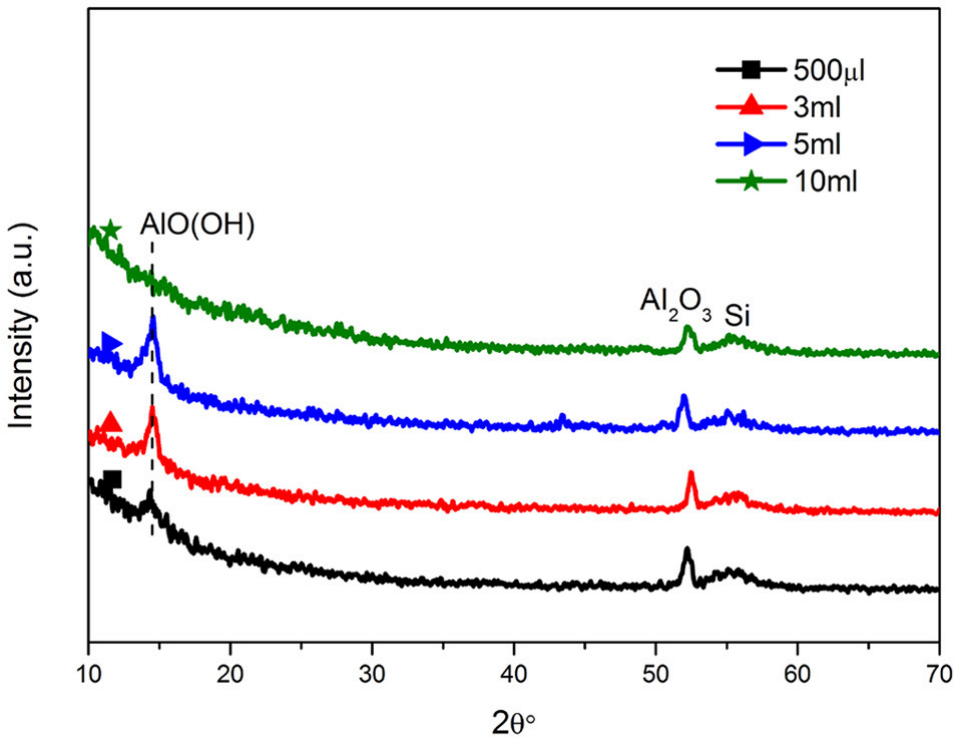
XRD diffraction patterns of the AOFs.

Figure 2 showed the XRD patterns of all samples. It was clear that all samples had the same phases. The diffraction patterns of all at 14.5° could be indexed to the orthorhombic γ-AlOOH phase (Lu et al., 2009), indicating that the crystalline boehmite was prepared under the hydrothermal condition. It showed that the peak at 14.5° of 10 ml sample was weaker than other samples, maybe because that an incompact layer of the upper nanowires and the lower nanohole (Figure 1J; Kim et al., 2012). The peaks at 2θ 52.2° showed Al_2_O_3_ (Shon et al., 2011; Zhong et al., 2011), proving that all ingredients were clear and stable. Because of the water content different in hydrothermal reaction, different degree of separation of the hydroxyl group, the 500 μl-AOF, 3 and 5 ml-AOF were composed by bohemite and alumina. However, the 10 ml-AOF was mainly composed of alumina.

**Figure 3:**
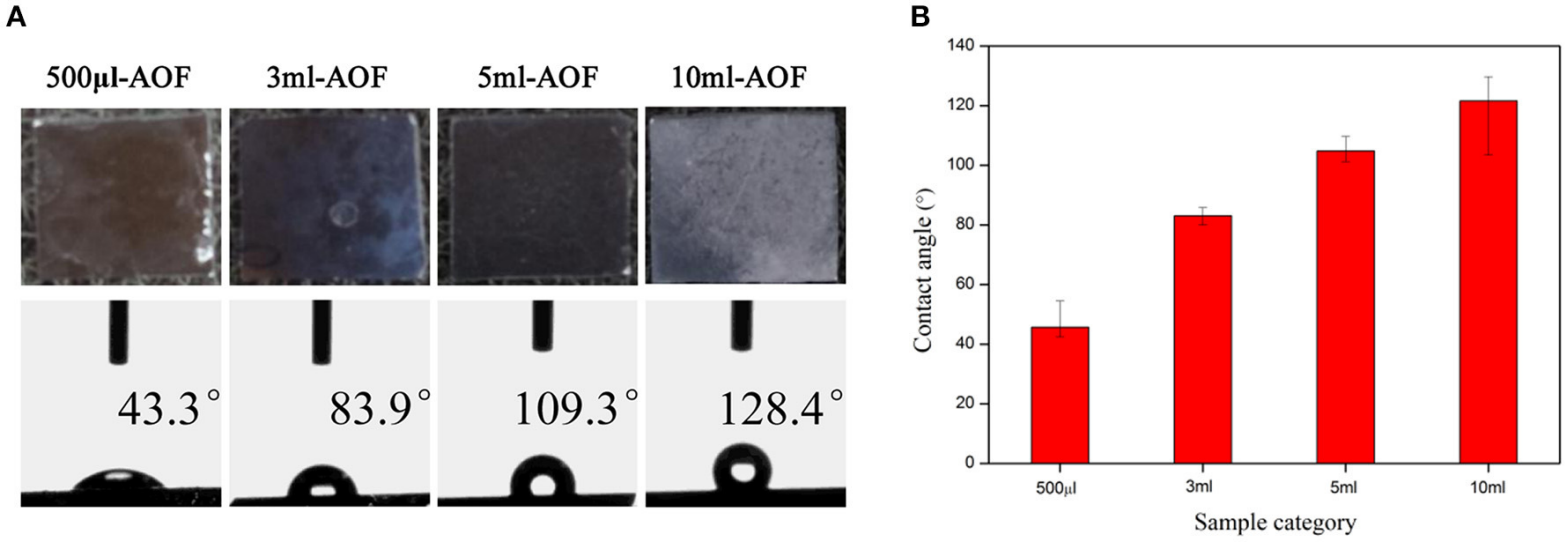
**(A)** Digital photos and water contact angles (are average of all contact angles to show the overall trend) of AOFs on Si substrates; **(B)** The tendency of water contact angles for the all samples.

The wetting (or non-wetting) properties can be produced by modifying the surface energy and surface roughness, which is lower than that of water for hydrophobic surfaces and higher for hydrophilic ones (Ohkubo et al., 2010; Park et al., 2010; Kim et al., 2012). In order to analysize the wetting or nonwetting properties of the films, the WCA were measured. The surfaces topographies and WCA of all AOFs were obviously different (Figure 3A). The water contact angles for 500 μl-AOF were below 90° due to the high surface energy caused by the gaps of AOF, suggesting that some AOFs were hydrophilic. As the water content increased, the hydrophobicity also scaled up, as shown in Figure 3B. The 10 ml-AOF sample was excellent hydrophobicity with the contact angle of 128.4°. It may be attributed to the upper nanowires and lower nanohole structures like some hills shown in SEM images of AOFs (Figure 1J). The hills, holding water on the surface, play the vital role in hydrophobic properties comparable to lotus effect (Samaha et al., 2012; Wen et al., 2017).

**Figure 4:**
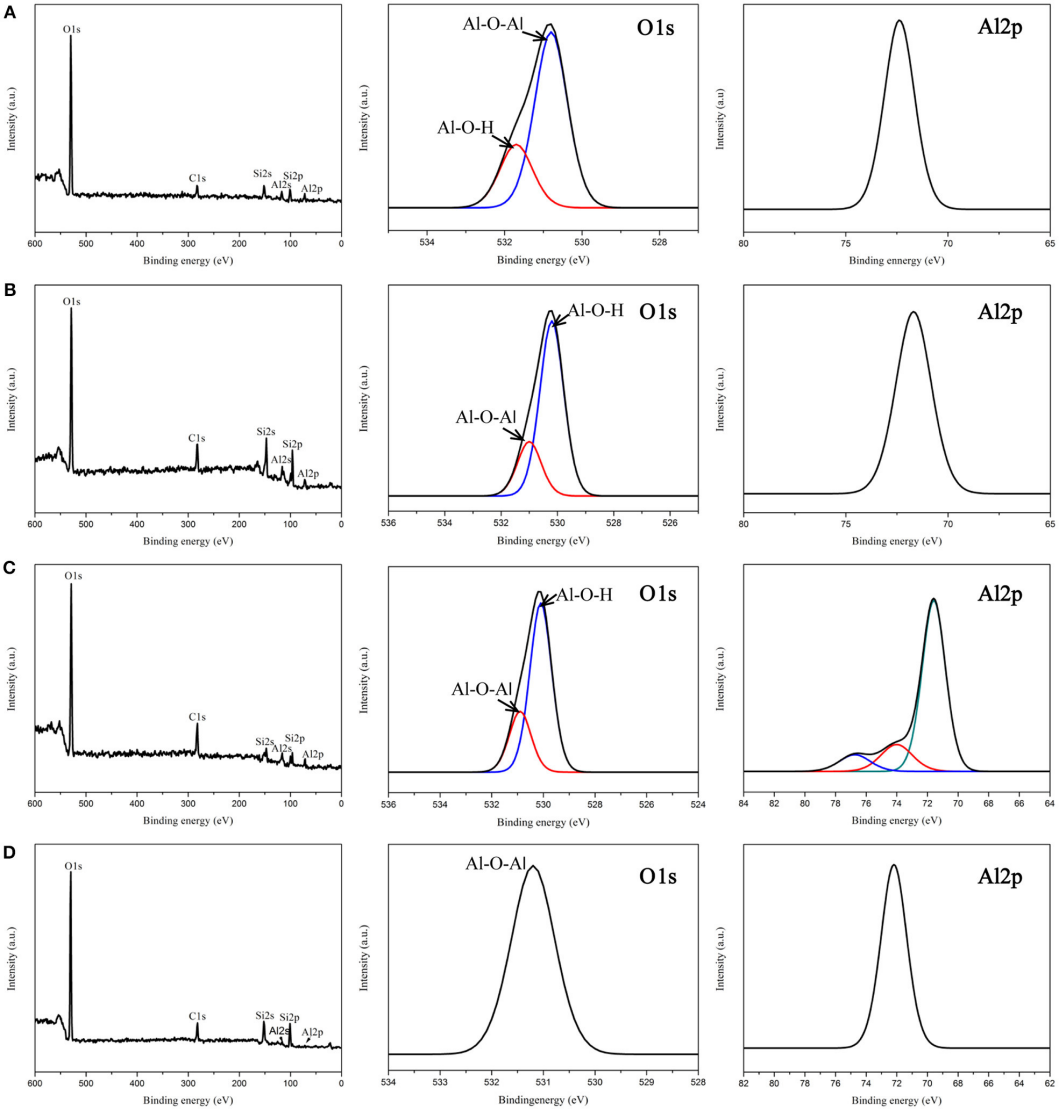
XPS spectrum of the aluminum oxide films: **(A)** 500 μl-AOF; **(B)** 3 ml-AOF; **(C)** 5 ml-AOF; **(D)** 10 ml-AOF.

Chemical and structural analyses of all samples were performed using XPS in Figure 4. As expected, Figures 4A–D revealed the presence of Al, O, Si, and C elements in all films. The O1s binding energies peaks of the 500 μl-AOF, 3 and 5 ml-AOF showed the two different oxygen species: a binding energy of 531.1eV is from the crystal structure (Al-O-Al); the AlOOH contains hydroxyl groups (Al-O-H). The O1s spectrum of 10 ml-AOF with 531.1 eV could be described accurately using only one component corresponding to Al-O-Al (Wang et al., 2009). The result was consistent with XRD analysis showed that the 10 ml-AOF contained Al2O3 and weaker hydroxyl groups. This may be because the increased water content in the hydrothermal reaction leads to the removal of the hydroxyl group. Moreover, the Al 2p peaks of 500 μl-AOF, 3 and 10 ml-AOF were single symmetrical component corresponding to Al(III). The 5 ml-AOF sample consisted of three peaks at 71.6, 74.0, and 76.7 eV corresponding to Al(III) and Al_2_O_3_ (Figure 4C; Tiwari et al., 2011).

**Figure 5:**
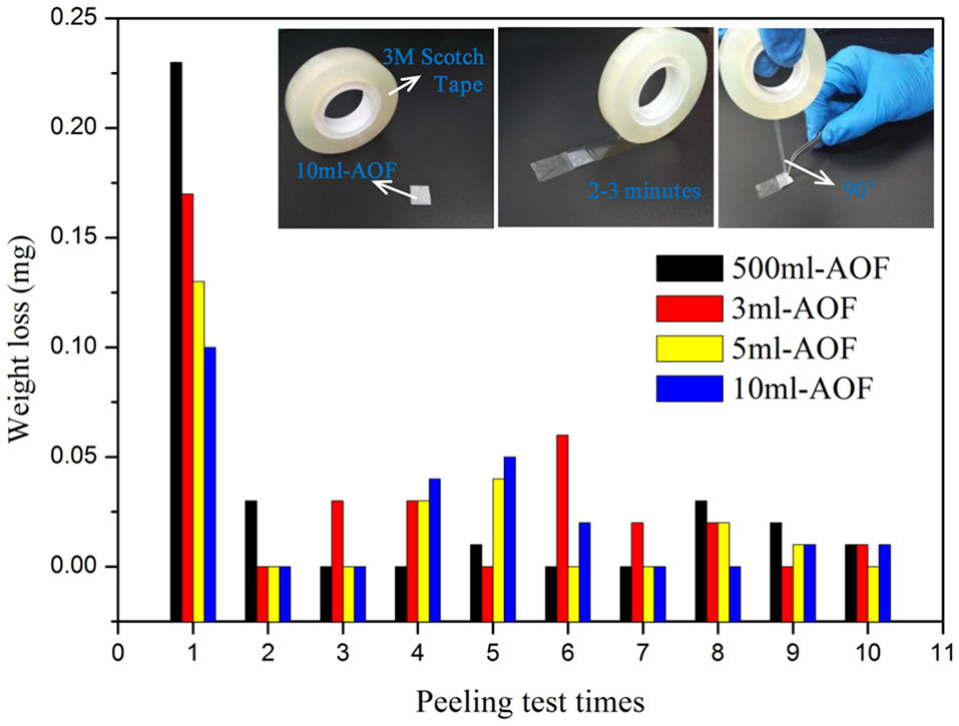
Adhension force test of AOFs by 3M peeling test, insert is the digital image of 3M peeling test.

In this study, the 3M peeling test was used to study the mechanical adhesion of AOFs (Arrowsmith, 1970; Lee et al., 2013; Wei et al., 2015). With the water content increased, it was clearly shown that films adhesiveness with substrates was increased after 1 peeling time (Figure 5). The 500 μl-AOF was easily removed from substrate, while the 10 ml-AOF could be stably on substrate. It illustrate that the nanowires structures make more contribution to the mechanical adhesion of films. However, during 2–10 peeling times, all samples no visible difference in the weight loss was observed before and after the peel-off test. Moreover, it was worth mentioning that all weight loss ratio of total sample was 0.1–0.19%. The result indicated that the AOFs had strong adhesiveness and mechanical stability.

**Figure 6:**
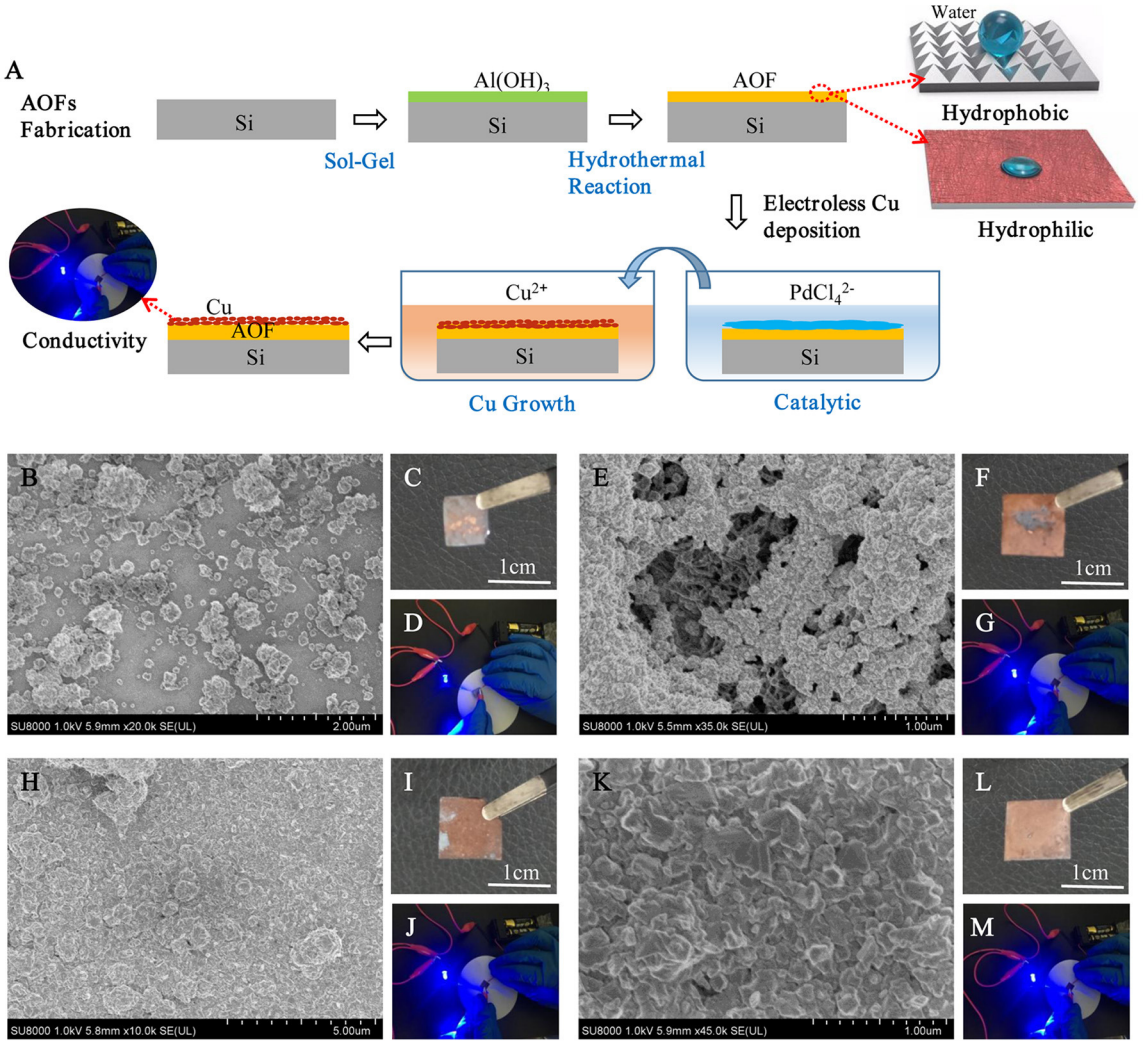
**(A)** Schematic illustration for the formation of electroless Cu deposition on the AOFs. SEM sans of the electroless deposition of Cu on AOFs, photographic images of Cu on different substrates and circuits to light a LED bulb by using a AOF with such patterned Cu coating as a connection part: **(B–D)** 500 μl-AOF; **(E–G)** 3 ml-AOF; **(H–J)** 5 ml-AOF; **(K–M)** 10 ml-AOF.

**Figure 7:**
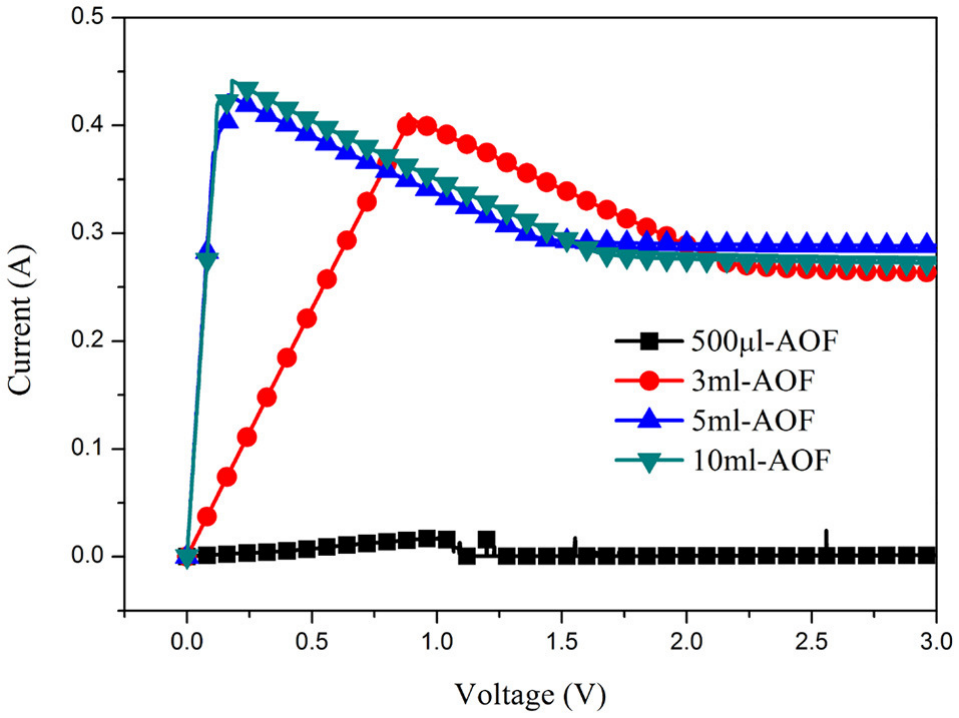
I–V curves of all AOFs with Cu coatings.

Figure 6A was the schematic illustration for the whole process of AOFs fabrication and electroless Cu deposition on the AOFs. The electroless Cu deposition on AOF coating could improve the conductivity of conductor device on the base of AOF insulation layer. The process was simple and easy to operate, using AOFs hydrophobic properties of application in many fields. In catalytic process, the positive charges on the AOFs generated an abundant adsorption of negative ${\text{PdCl}}_{4}^{2-}$ catalyst layer to achieve uniform metal deposition with good adhesion properties, then immersed in the Cu electroless bath to grow Cu (Wang et al., 2016). In order to study morphology and conductivity for electroless Cu deposition, SEM micrographs, photographic images, and circuits to light a light-emitting diode (LED) bulb were illustrated in Figures 6B–M. The Figure 6B showed the morphology of Cu coating on 500 μl-AOF composed by some blocky structures. The Cu coatings on 3 and 5 ml were much tighter (Figures 6E,H). When electroless deposition of Cu is on the 10 ml-AOF, the SEM scans of Cu coating were closely connected by blocks without any gaps (Figure 6K). With the water content increasing in the fabrication of AOFs, the SEM images of Cu coatings were gradually dense and flat, which agreed well with the digital photographs in Figures 6C,F,I,L). Thus, AOFs play an important role in electroless Cu deposition. Owing to the superior conductivity, Cu coatings on AOFs can be easily used in circuits which integrate LED, as shown in Figures 6D,G,J,M. Figure 7 showed the I–V curves and conductance of the Cu coatings on AOFs. For the 500 μl, the I–V showed low conductivity. However, the 5 and 10 ml-AOF showed higher current for aluminum oxide with Cu than 3 ml and 500 μl due to integrity of Cu coatings. Obviously, the excellent performance of Cu coating is mainly attributed to the structure of AOF, while the compactness of Cu coating on AOFs plays a leading role in forming excellent electrical conductivity.

## Conclusions

In this paper, AOFs can be synthesized using the sol-gel method by magnetic stirrer and hydrothermal reaction. As the water content increases in hydrothermal reaction, the hydrophobicity of AOFs also proportionally increase. The water contact angle of 10 ml-AOF is 129.3°. Adding 10 ml deionized water leads to the formation of the upper nanowires and the lower nanohole. The structure may be responsible for hydrophobicity of AOFs. Furthermore, the conductivity of electroless Cu deposition on 10 ml-AOF is the best. This work reveals a new sol-gel method by magnetic stirrer, which is simple and easy to operate. By adding different water content in PPL hydrothermal synthesis reactor, the AOFs with the best hydrophobic properties can be obtained.

## Author contributions

CF and XZ designed and guided this study. MP designed the research, performed the aluminum oxide films fabrication, and conducted the chemical synthesis. WL performed the electroless Cu deposition. MP, LP, and CW participated in the electroless Cu deposition. MP and XZ analyzed the data and wrote the manuscript. All authors approved the final version.

### Conflict of interest statement

The authors declare that the research was conducted in the absence of any commercial or financial relationships that could be construed as a potential conflict of interest.
